# From Foreground to Background: How Task-Neutral Context Influences Contextual Cueing of Visual Search

**DOI:** 10.3389/fpsyg.2016.00852

**Published:** 2016-06-07

**Authors:** Xuelian Zang, Thomas Geyer, Leonardo Assumpção, Hermann J. Müller, Zhuanghua Shi

**Affiliations:** ^1^China Centre for Special Economic Zone Research, Research Centre of Brain Function and Psychological Science, Shenzhen UniversityShenzhen, China; ^2^General and Experimental Psychology, Department of Psychology, Ludwig-Maximilians-Universität MunichMunich, Germany; ^3^Department of Psychological Science, Birkbeck, University of LondonLondon, UK

**Keywords:** contextual cueing, foreground-background segmentation, 3D visual search, spatial memory, implicit learning, grouping

## Abstract

Selective attention determines the effectiveness of implicit contextual learning (e.g., Jiang and Leung, 2005). Visual foreground-background segmentation, on the other hand, is a key process in the guidance of attention (Wolfe, 2003). In the present study, we examined the impact of foreground-background segmentation on contextual cueing of visual search in three experiments. A visual search display, consisting of distractor ‘L’s and a target ‘T’, was overlaid on a task-neutral cuboid on the same depth plane (Experiment 1), on stereoscopically separated depth planes (Experiment 2), or spread over the entire display on the same depth plane (Experiment 3). Half of the search displays contained repeated target-distractor arrangements, whereas the other half was always newly generated. The task-neutral cuboid was constant during an initial training session, but was either rotated by 90° or entirely removed in the subsequent test sessions. We found that the gains resulting from repeated presentation of display arrangements during training (i.e., contextual-cueing effects) were diminished when the cuboid was changed or removed in Experiment 1, but remained intact in Experiments 2 and 3 when the cuboid was placed in a different depth plane, or when the items were randomly spread over the whole display but not on the edges of the cuboid. These findings suggest that foreground-background segmentation occurs prior to contextual learning, and only objects/arrangements that are grouped as foreground are learned over the course of repeated visual search.

## Introduction

In everyday life, we constantly receive a massive amount of sensory input that would require an unrealistic amount of cognitive resources to be all processed. To ensure the functioning of higher-level mental processes, we benefit from sophisticated attentional mechanisms that help us select and process information that is important, and deselect information that is unimportant, for performing relevant tasks and ongoing actions (Treisman and Gelade, 1980; Wolfe, 2003). To illustrate, imagine a situation in which one searches for a car in a parking lot: search strategies would be different depending on whether one searches for a car in a global scene context (e.g., searching on the east side of the parking deck) or in a local configural context (e.g., searching for a car parked between two cars of the same model/brand). The specific ‘contexts’ in these scenarios would determine how and where attention should be deployed, thus ‘saving’ cognitive resources by processing only the most relevant information to the task at hand.

The interplay between scene-based and configuration-based context has been investigated in a number of studies using the ‘contextual-cueing’ paradigm (e.g., Brockmole and Henderson, 2006; Brooks et al., 2010; Kunar et al., 2013; Rosenbaum and Jiang, 2013). In the standard contextual-cueing task (e.g., Chun and Jiang, 1998; Chun, 2000; Pollmann and Manginelli, 2009a; Geringswald et al., 2012; Annac et al., 2013; Geyer et al., 2013), participants search for a ‘T’-shaped target amongst ‘L’-shaped distractors. Unbeknownst to participants, half of the search displays are repeatedly presented, that is: ‘old’, displays, in which the locations of both the target and the distractors are kept constant across trials (though with target identity being variable), while the other half of search displays presents novel items arrangements. In more detail, in these ‘new’ displays, the distractors change locations randomly across trials, while the target locations are nevertheless controlled to equate target location repetition between old and new displays. The common finding is that reaction times (RTs) are faster to targets in old compared to new spatial arrangements, an effect referred to as ‘contextual cueing’. Interestingly, when participants are asked about display repetitions in an explicit old-display recognition test at the end of the search experiment, they are typically unable to discriminate old from new displays to a level better than chance. This has led to the idea that contextual cueing is an implicit-memory effect, though the role of consciousness in contextual cueing has become a controversial issue recently (for a review, see Vadillo et al., 2015).

Since the seminal study of Chun and Jiang (1998), the contextual cueing paradigm has proven to provide a powerful tool in the investigation of visual search and attention. An important issue in the present context concerns whether contextual cueing is itself influenced by attention. Regarding this question, it has been proposed that perceptual segmentation – or visual grouping – regulates the acquisition of contextual memory traces. For example, some studies suggested that contextual cueing is determined by spatial grouping, evidenced by findings that only display items in the vicinity of the target are effectively acquired in contextual learning (e.g., Olson and Chun, 2002; Brady and Chun, 2007; Zang et al., 2015). Other findings (e.g., Brockmole et al., 2006; Kunar et al., 2006; Shi et al., 2013), by contrast, provide strong evidence in favor of the idea that global context is necessary for the cueing effect to occur (i.e., the observers form associations between the target and the entire distractor background). Discrepancies also arise in relation to featural grouping (e.g., Jiang and Chun, 2001; Jiang and Leung, 2005). On the one hand, under conditions in which the search items could be grouped based on common color (e.g., groups of black vs. white items), Jiang and Leung (2005) observed contextual cueing only when the distractors, as well as the target, in a specific (e.g., the white) color group appeared at identical locations (the locations of the distractors in the other color group, e.g., black distractors, were either maintained constant or varied) – suggesting that, by ‘default’, contextual target-distractor associations are formed within individual color groups (see also Geyer et al., 2010). Note that observers in Jiang and Leung’s (2005) study were explicitly instructed to search for a target defined by a pre-specified color (e.g., white), invoking a feature-based attentional set. Interestingly, the magnitude of the cueing effect in this ‘attended-old’ condition was comparable to cueing in a ‘both-old’ condition in which all, black and white, distractors appeared at identical locations. On the other hand, when presenting the distractors in different – ‘small’ and, respectively, ‘large’ – sizes, Conci et al. (2011) found contextual cueing to be reduced in the ‘grouping’ condition compared to the ‘standard’ condition in which all distractors were of the same size. This suggests that feature-based attention (to one or the other group of items) might even hamper contextual cueing. Thus, although manipulation of display features (e.g., color, size) does provide a promising tool for investigating the role(s) of feature-based attention and grouping for contextual cueing, the evidence available to date is rather mixed.

Findings from other studies that investigated attentional constraints in relation to scene context complicate the picture of the link between attention and contextual cueing even further. For instance, Brooks et al. (2010) examined contextual cueing in visual search arrays that were presented on the surface of a green ‘table’ located in the center of a real-world scene display, where the repetition of search array configurations and scene displays were manipulated independently. In this condition, both the configuration of the search items and the real-world scene (or, alternatively, either one but not the other) could in principle act as context cues for the search target. The results revealed a contextual cueing effect only in the ‘constant-configuration/variable-scene’ condition, but not in the ‘variable-configuration/constant scene condition’, which led Brooks et al. to propose a ‘configuration-dominant’ influence in contextual cueing. Nonetheless, a ‘scene-dominant’ effect was reported by Rosenbaum and Jiang (2013) when they presented the visual search display across the entire scene, including both central (foveal) and peripheral item locations. Participants were first trained on predictive displays containing both a scene and a search array configuration (i.e., the target location was consistently associated with the same scene and the same search array configuration), and then were tested with two types of search displays: a scene-predictive display, in which the target location was associated with the same scene but embedded in a different search array, and an array-predictive display, in which target location was associated with the same search array but a different scene. The results revealed reliable contextual cueing when the scene, but not array, was predictive, arguing in favor of a more important role for scene-based, as compared to configuration-based, context in contextual learning.

While in one hand the studies reviewed above generally support the idea that contextual learning is subject to perceptual constraints, on the other hand they merely focused on the (relative) extent to which the acquisition of contextual cues is influenced by certain visual properties. Arguably, however, in addition to producing equivocal findings, these studies failed to provide a general view as to how spatial associations are formed in the first place, that is: what are the principles that determine the learning of target-distractor associations (e.g., configuration- vs. scene-based contextual cueing)? Here, we propose that spatial context learning is constrained by a more basic, yet fundamental process, namely: ‘foreground-background segmentation’, which governs how attention is deployed. Foreground-background segmentation has been shown to occur quite early in visual processing, prioritizing the foregrounded ‘candidate’ perceptual units for further processing (e.g., Baylis and Driver, 1992, 1993; Driver et al., 2001; Mazza et al., 2005). Accordingly, attention is biased toward the selected foreground, yielding an enhanced representation (and learning) of foregrounded items (Mazza et al., 2005). Importantly, processes of foreground-background segmentation are not limited to the search items, but rather involve the entire visual scene. On this view, determining the role of foreground-background segmentation may provide a unified account as to how grouping, scene- and configuration-based information, influences contextual cueing in visual search (Brockmole et al., 2006; Brooks et al., 2010; Kunar et al., 2013; Rosenbaum and Jiang, 2013). In a nutshell: we propose that the information that is selected as foreground determines the contextual cueing effect.

**FIGURE 1:**
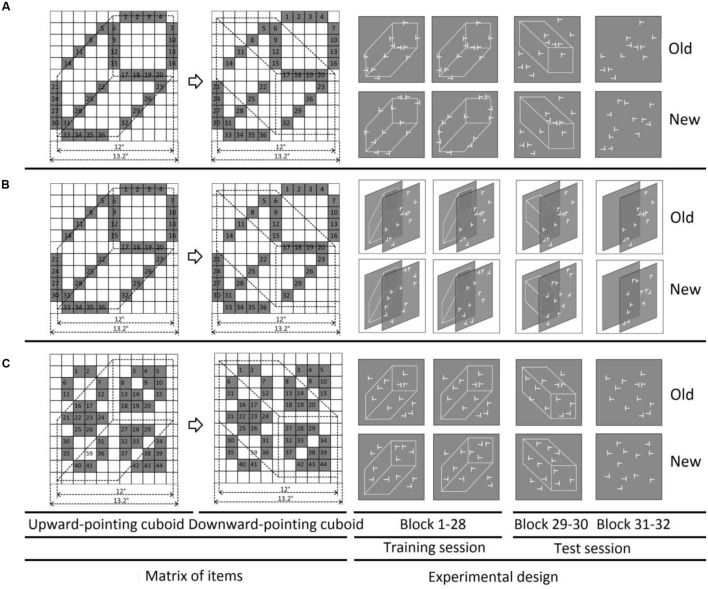
**Stimulus configurations and schematic paradigm used in Experiments 1, 2, and 3**. Left: possible positions (gray grids) for search items in three experiments, respectively, (for both ‘upward-pointing’ and ‘downward-pointing’ cuboid). The visual search items were presented on the edges of the pseudo cuboid on the same plane in Experiment 1 **(A)**, while projected on the edges of the pseudo cuboid, but presented on the different planes in Experiment 2 **(B)**, and on the space location but not edges of the ‘upward-pointing’ and ‘downward-pointing’ cuboid in Experiment 3. The grids, numbers, and the gray color were invisible during the actual experiments. The whole display subtended as 13.2 × 13.2°. Right: **(A)** schematic illustration of three sessions used in Experiment 1: the training session (block 1–28), the first test session (block 29–30), and the second test session (block 31–32). For the old item-based configurations, each target was paired with a particular consistent distractor sets, repeated once per block; while for the novel item-based configuration, the target was paired with newly generated distractor sets for each presentation. The task-neutral cuboid was the same for both old and new displays, ‘upward-pointing’ during the training session, ‘downward-pointing’ during the first test session, and absent during the second test session. **(B)** Schematic illustration of Experiment 2. The visual stimuli used in Experiment 2 were the same as used in Experiment 1, except the pseudo cuboid was presented on the deeper depth plane, separated from the search items on the front plane. The schematic illustration was plotted from a -50° of view angle in order to show the depth information well. In the real experiment participants wore 3D glasses (Optoma ZF2100) and viewed the display in front of the visual search items, such that the search items were still on the edges of the cuboid, though in separated planes. **(C)** Schematic illustration of the three sessions in Experiment 3.

In order to validate our hypothesis, the current study investigated attentional constraints on context learning by examining scene-based interference in a conventional visual search task. In order to separate grouping effects from context learning, we conducted two experiments presenting visual search items (‘T’ and ‘L’s) together with a task-neutral (i.e., irrelevant for deciding on the required search response) context (e.g., a 2D projection of a 3D cuboid, see **Figure 1**) that was not predictive of the target location. The reason for choosing the cuboid as a task-neutral object was twofold: First, a cuboid object is ultimately larger and more salient than the individual search items. It serves as a ‘global shape’ stimulus, enabling us to examine for the (novel) effects of global, 3D stimulus attributes on contextual cueing, in addition to the effects of semantic context (Brockmole and Henderson, 2006) or color context (Jiang and Leung, 2005; Geyer et al., 2010). Second, in the real world, visual search operates in 3D environments, and the learning of visual contexts could interact with 3D objects that may exist in the scene. Therefore, a task-neutral cuboid enables us to investigate the interactions between 2D items and 3D objects in contextual cueing. In Experiment 1, all visual search items were located on the edges of the cuboid, ensuring that the shape of the cuboid could be easily picked up as foreground information. In Experiment 2, by contrast, the cuboid was assigned the role of background by virtue of being presented on a different, distant depth plane to the search items. In Experiment 3, the visual search items were randomly spread over the whole display but not on the edges of the cuboid (e.g., with a weak association between items and the cuboid), thus assigned the cuboid to the background during visual search. Following an initial training session, in the test sessions, the cuboid was either rotated or entirely removed to examine for possible effects of figure-ground segmentation on contextual cueing.

Our hypothesis was that foreground context would play a more important role in contextual guidance than background context, that is, the cuboid would influence contextual learning in Experiment 1 (in which, during learning, observers were unable to separate the cuboid and the search items), but not in Experiments 2 and 3 (in which depth segmentation was possible, or associations between search items and cuboid were weak, permitting the arrangement of the search items to be learned without reference to the cuboid object). Accordingly, we expected a decrease, if not complete abolishment of the contextual cueing effect after the change (or removal) of the ‘foreground’ cuboid at the transition from training to test/transfer in Experiment 1, but not in Experiments 2 and 3. Alternatively, if processes of foreground-background segmentation do not affect contextual cueing, presenting the cuboid as foreground during learning (i.e., in Experiment 1) should not modulate contextual cueing in the subsequent test session.

## Experiment 1

Experiment 1 investigated whether a task-neutral ‘cuboid’ context interpreted as foreground would be encoded in the memory representation underlying contextual cueing. Crucially, we examined whether an already acquired context (in the learning phase of the experiment) would still be used after a change or complete removal of the task-neutral cuboid in the test phase. To this end, the search items were randomly arranged on the edges of the cuboid (see **Figure 1** for an example) such that the frame of the cuboid and the search items would be automatically co-located, or linked with each other, in the visual space. As shown by previous studies (Palmer, 1992; Palmer and Rock, 1994; Han et al., 1999), uniform connectedness is a strong factor in perceptual grouping and organization, occurring at a very early stage. Therefore, the task-irrelevant cuboid was expected to be grouped together with the task-relevant visual search items and, thus, be interpreted as foreground context.

### Materials and Methods

Although contextual cueing is a stable effect observed repeatedly in previous studies (e.g., Chun, 2000; Goujon et al., 2015), it is important to note that some 30% of the participants may reveal from none to negative contextual cueing (Schlagbauer et al., 2012). As our aim was to examine how the change of the task-neutral cuboid affects contextual cueing, it was crucial to limit investigation of the transfer effect to only those participants who had already learned, and displayed a positive cueing effect in response to, the original (‘old’) displays before the cuboid variation. Since Experiment 1 (as well as Experiments 2 and 3) consisted of two stages, only those participants who exhibited a positive contextual cueing effect in the first, training stage continued on to the second test stage (for the other participants, the experiment was terminated after the training stage). Two criteria were used to identify positive cueing effects: the grand mean response times (RTs) over the whole training session and the mean RTs for the last epoch (see definition of ‘epoch’ below) had to be faster for old compared to the new displays. This procedure has been used routinely in many other studies investigating transfer effects of contextual cueing (Conci et al., 2011; Conci and Müller, 2012; Zellin et al., 2013a,b).

#### Participants

Eleven participants (eight females, mean age: 26 ± 4.54 years) took part in the training session, ten of whom (seven females, mean age 26.5 ± 4.45 years old) went on to complete the test session. Participants were paid 8 Euro per hour for their participation. The experiment was approved by the ethics committee of the Department of Psychology of LMU Munich.

#### Apparatus and Stimuli

The experiment was conducted in a sound-attenuated, dimly lit cabin (2.95 cd/m^2^). The visual displays were presented on a 21-inch LACIE CRT monitor, with a refresh rate of 100 Hz. The viewing distance was set at 57 cm, and kept constant with the use of a chin rest. The search displays comprised of 12 search items (each 0.8° × 0.8° of visual angle in size and 24.24 cd/m^2^ in luminance; the display background was gray: 6.33 cd/m^2^), consisting of one ‘T’-shaped target and eleven ‘L’-shaped distractors. Similar to previous studies (Jiang and Chun, 2001; Olson and Chun, 2002; Zang et al., 2015), the ‘L’ distractors had a small offset (0.12°) at the line junctions to make them more similar to the target ‘T’. The task-neutral object was a ‘pseudo’ cuboid (i.e., a cuboid projected onto a 2D plane, extending 12° × 12° of visual angle; see **Figure 1** for an example), composed of nine white lines (24.24 cd/m^2^). Two cuboid orientations, ‘upward-pointing’ and, respectively, ‘downward-pointing’, were used for the training and test sessions, respectively. The ‘square’-face of the upward-pointing cuboid was located in the upper-right quadrant, while the downward-pointing cuboid was created by rotating the (upward-pointing) cuboid 90° clockwise, so as to position the square face in the bottom-right quadrant (see **Figure 1**).

For each search display, the ‘L’ distractors were randomly rotated 0°, 90°, 180°, or 270° from the vertical midline, while the ‘T’ target was rotated 90° either clockwise or counter-clockwise, pointing to the right or to the left (and requiring a ‘left’ or, respectively, ‘right’ response). Both ‘T’ and ‘L’s were randomly placed at 36 possible locations inside an invisible 11 × 11 grid square area, with each location subtending 1.2° × 1.2° of visual angle. The 36 possible locations were selected on the edges, but not the vertices, of the trained cuboid (see left in **Figure 1A**). In this way, the position of the cuboid was strongly linked to the positions of the search items.

#### Procedure and Design

Participants were asked to discriminate the orientation of the target letter ‘T’ as fast and accurately as possible by pressing either the left or the right arrow key on the keyboard, using their left- and right-hand index fingers, respectively. Each trial started with the presentation of a central fixation cross for 800–1000 ms, which was immediately followed by a search display. The search display remained on the screen until a response was made or (in the absence of a response) until 10 s had elapsed. The next trial started automatically after a random interval of 1.0–1.2 s. As illustrated in **Figure 1A**, the experiment consisted of a 28-block training session, two 2-block test sessions, and a 3-block recognition session. Each block of 16 trials contained 8 old and 8 new displays, randomly intermixed. As for displays generation, for each participant, 16 possible target locations were generated; eight target locations for old and eight target locations for new displays. For old displays, except for target’s orientation, both target and distractor locations were kept constant across blocks, whereas for the new displays only target locations (except orientation) were kept constant. By maintaining target locations constant in both old and new displays we equate target location repetition effects between these displays.

During the training session, an upward-pointing cuboid, with search items presented on its edges (but not vertices), served as the task-neutral scene for both old and new displays. Since the very same cuboid was shown on each trial, it could not cue the target location in any better way for the old compared to the new displays. Therefore, any differences in RT performance between the old and new displays were attributable solely to either the configural context of the search items, or the interaction between the task-neutral cuboid and the search items. The cuboid was rotated by 90° in clockwise direction in the subsequent test session, in both old and new displays, while the configural context of the search items (old displays) was held constant across the two sessions. With this variation, most of the visual search items (more than 88%) were no longer located on the edges of the rotated (downward-pointing) cuboid, thus clearly disrupting any spatial association between the task-neutral cuboid and the search array. In the second test session, the cuboid was entirely removed from the search display.

Once the search task was completed, participants performed three consecutive blocks of recognition trials, with an ‘upward-pointing’ cuboid, a ‘downward-pointing’ cuboid, and ‘no cuboid’, respectively. Participants were told that half of the displays were repeated displays from the search task, and their task was to decide whether or not they had already seen a given display in the previous search task (by pressing the left and right arrow keys to respond ‘yes’ and ‘no’, respectively). The display presentation lasted maximum of 20 s (i.e., twice as long as the 10 s in the search sessions).

Prior to the experiment, participants practiced the experimental task with upward-pointing cuboids in one block of 16 trials. Only new display configurations were shown during practice. Participants were allowed to take a break between blocks of the experiment.

### Results

#### Search Task

**FIGURE 2:**
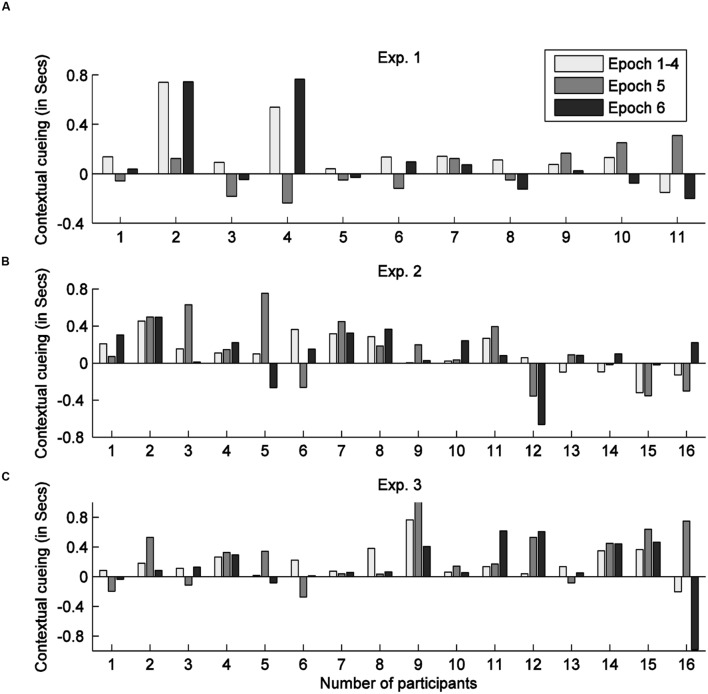
**Contextual cueing scores (RT differences between the new and old display) in the test session for individual observers in Experiments 1 (A), 2 (B), and 3 (C) respectively**.

**FIGURE 3:**
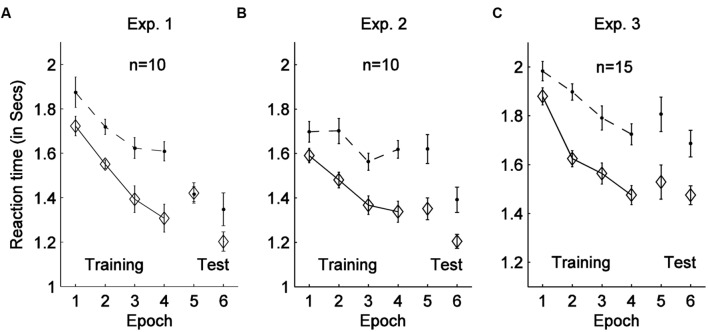
**Mean RTs with associated standard errors are shown as a function of experimental epoch and display context (old, indicated by solid-diamond lines, vs. new, indicated by dash-dot lines) for Experiments 1 (A), 2 (B), and 3 (C)**. Epochs 1–4 were in the training session, while epoch 5 and 6 were the test sessions with rotated or removed cuboid.

The data of all 11 participants (see **Figure 2**) were analyzed together for the training session, and of the 10 participants who completed the whole experiment for the test and recognition sessions. Each 7 consecutive blocks in the training session were grouped into one ‘epoch’, forming 4 training epochs, and each test session (two blocks) was grouped into one epoch, forming epoch 5 (hereafter referred to as ‘test session I’) and epoch 6 (‘test session II’), respectively. The mean RT of the 10 positive cueing learners with epochs and contexts as factors are shown in **Figure 3**.

Trials with erroneous responses or ‘outlier’ RTs shorter than 200 ms and longer than 3 SDs above the mean were excluded from further analyses. Both the overall mean error and outlier rates of the training session were low (mean error rates: 1.00%; outliers: 2.27%). Note that the error/discard rates of the positive cueing learners were even lower in the test session (<1.00%; a similar result was also observed in Experiments 2 and 3). The error rates were comparable across all conditions: context, *F*(1,10) = 2.34, *p* = 0.16, $\eta_{p}^{2}$ = 0.19, epoch, *F*(3,30) = 1.47, *p* = 0.24, $\eta_{p}^{2}$ = 0.13, and interaction, *F*(3,30) = 1.79, *p* = 0.08, $\eta_{p}^{2}$ = 0.20. That is, accuracy did not improve significantly, for any of the context conditions (old, new displays) over the training session.

Examining training performance of all participants recruited in the experiment, a 2 × 4 repeated-measures ANOVA on RTs with the factors context (old, new displays) and epoch (1–4) revealed significant main effects of context [*F*(1, 10) = 5.89, *p* < 0.05, $\eta_{p}^{2}$ = 0.37] and of epoch [*F*(1.48, 14.75) = 20.86, *p* < 0.01, $\eta_{p}^{2}$ = 0.68], as well as the context × epoch interaction [*F*(3,30) = 3.75, *p* < 0.05, $\eta_{p}^{2}$ = 0.27]. RTs were overall 180 ms faster for old compared to new displays, and 329 ms faster in epoch 4 compared to epoch 1. The interaction indicated that contextual cueing developed over the course of training. Additional *post hoc* tests confirmed that the contextual cueing effect reached significance in epochs 3 and 4 (*p* < 0.05), but not in epochs 1 (*p* = 0.27) and 2 (*p* = 0.18). Taken together, these results are indicative of both procedural learning, indexed by a general speeding-up of task performance across epochs (in all conditions), and contextual learning, that is, a RT advantage for old versus new displays, over the training session.

In the subsequent test sessions, the mean RTs of the ten positive cueing learners (in the training session) appeared somewhat faster for ‘old’ compared to ‘new’ displays. However, this numerical difference was neither significant in epoch 5 (test session I) nor in epoch 6 (test session II), as indicated by paired-sample *t*-tests: epoch 5, *t*(9) = 0.09, *p* = 0.93; epoch 6, *t*(9) = 1.4, *p* = 0.20. Additional JZS Bayes Factor (BF) analysis (Rouder et al., 2009) revealed a BF of 4.29 for epoch 5 and of 1.85 for epoch 6. According to Jeffries (1961), a value greater than 3 provides solid evidence for the null hypothesis. Therefore, the result patterns in the two test sessions favor the null hypothesis (despite a non-significant trend for contextual facilitation in epoch 6). Thus, in summary, the results of the test sessions suggest that, although the cuboid itself was not predictive of the target location, it was nevertheless encoded in the representation driving contextual cueing. As a result, when the aspect of the foreground cuboid was changed (test session I) or when the cuboid was entirely removed (test session II), contextual facilitation was effectively abolished.

To examine the effect of cuboid change on RT performance, a further 2 × 3 repeated-measures ANOVA was performed for the last two training blocks and the test sessions, with context (old, new) and session (training, test session I, test session II) as factors. The results revealed no significant context effect, *F*(1, 9) = 3.15, *p* = 0.11, $\eta_{p}^{2}$ = 0.26, but a significant session effect, *F*(2,18) = 9.42, *p* < 0.05, $\eta_{p}^{2}$ = 0.51, and the context × session interaction was significant, *F*(2,18) = 3.94, *p* < 0.05, $\eta_{p}^{2}$ = 0.31, the latter confirming the above finding that contextual cueing decreased significantly from the training to the test sessions: mean RTs to new [old] displays were 1.68 [1.30] s, 1.42 [1.42] s, and 1.35 [1.20] s with the upward-pointing (training session), the downward-pointing (test session I) and the no-cuboid condition (test session II), respectively. As it can be seen, however, the reduction of cueing was mainly due to responses to new displays being expedited in the test sessions compared to the training session [by 260 ms in test session I, *t*(9) = 2.63, *p* < 0.05; and by 328 ms in test session II, *t*(9) = 3.51, *p* < 0.01]. In contrast, for the old displays, responses became significantly faster (compared to training) only when the cuboid was removed (98 ms), *t*(9) = 2.63, *p* < 0.05, while tending to be slower when the cuboid changed (121 ms), *t*(9) = 1.57, *p* = 0.15. The RT facilitation for new displays suggests that the search task became easier with the rotated, downward-pointing cuboid (or without cuboid) compared to search with the original, upward-pointing cuboid object. In other words, detection of the target on the foreground cuboid may be difficult as such, as reflected in slower RTs. RTs are expedited, in turn, as soon as the cuboid is ‘pushed’ to the background (recall that after the change, more than 88% of the search items no longer appeared on the edges of the cuboid, facilitating segregation of the search items [foreground] from the cuboid [now background]). Interestingly, with the backgrounded downward-pointing cuboid, RTs was longer compared to the no-cuboid condition, suggesting that the downward-pointing cuboid still causes a cost in processing time – perhaps attributable to the demands associated with keeping the irrelevant background out of the search.

A similar expedition of responses would, in principle, also be expected for old; however, here the change or entire removal of the cuboid object, overlaid on a (relative to the training session) constant search item configuration, did affect the search performance. The net result would be that facilitation of responses due to (in the downward-pointing cuboid condition) improved or (in the no-cuboid condition) no longer necessary foreground-background segregation on the one hand and inhibition of responses due to partial cuboid-configural changes on the other would cancel each other out, effectively abolishing the contextual cueing effect in the test sessions. Thus, in summary, the results indicate that the foreground task-neutral cuboid was learned together with the spatial context during contextual learning, and the rotation or removal of the cuboid in the test sessions abolished a well-established contextual cueing effect.

#### Recognition Results

Trials with RTs exceeding 20 s (i.e., on which participants failed to respond in time) were excluded from the analysis; this led to the removal of 0.38% of the data. For the 10 positive cueing learners who finished the whole experiment (training, test, and recognition sessions), their mean hit rates (i.e., correctly identified old display as repeated) were 61.25, 53.75, and 60.54% in the three consecutive blocks, respectively, which were numerically higher than the false alarm rates (i.e., new display incorrectly judged as old; 57.05, 53.04, and 48.75%, respectively). However, these differences were not significant: first recognition block display including upward-pointing cuboid, *t*(9) = 0.67, *p* = 0.52, JZS Bayes Factor = 3.64; second block with display including downward-pointing cuboid, *t*(9) = 0.07, *p* = 0.95, JZS Bayes Factor = 4.29; third block without cuboid, *t*(9) = 1.34, *p* = 0.21, JZS Bayes Factor = 1.98. As the power of each single (block) test may have been too low to reveal a significant level of explicit recognition, following a criticism by Vadillo et al. (2015) leveled against many previous contextual-cueing studies, we collapsed the three recognition blocks together (to increase the statistical power): nevertheless, the results still revealed no significant effect: *t*(9) = 0.81, *p* = 0.44, JZS Bayes Factor = 3.18. Further participant-wise analysis failed to reveal a systematic correlation between the recognition performance (d’) in the collapsed recognition blocks and the magnitude of contextual cueing in the last two test blocks, *r* = 0.05, *p* = 0.89. Thus, taken together, there was no evidence that contextual cueing in the current experiment was based on explicit memory of old displays.

### Discussion

The major finding of Experiment 1 was that contextual cueing was abolished when the cuboid, serving as a task-neutral context, changed its orientation or was removed in the test session. Two alternative reasons could explain the loss of the contextual-cueing effect. The first is that the search-guiding contextual associations acquired during the training session were established with reference to the cuboid, despite the fact that the cuboid itself was completely non-informative with respect to the target location. In other words, the cuboid was perceptually foregrounded and encoded together with the distractor configuration during contextual learning (‘foreground-learning’ alternative). As a result, contextual cueing was sensitive to the change of the cuboid object. Alternatively, the absence of contextual cueing in the test session was due to the change of the cuboid object. In this case, contextual cues were learned only in relation to the configuration of the search items; however, retrieval of the learned context was blocked by the salient change of the display even though this change was task-irrelevant (‘blocked-retrieval’ alternative). The key difference between these two accounts is that the first, ‘foreground learning’, assumes that the foreground context, including the task-neutral cuboid, is learned in conjunction with the spatial-array context; by contrast, the second ‘blocked retrieval’ alternative, emphasizes that contextual memory is solely constructed based on the spatial-array, but the retrieval could be blocked by the variation of the cuboid. To further disassociate these accounts, we ‘weakened’ the spatial association between the search items and the cuboid object by placing them in separate depth planes in Experiment 2. We hypothesized that placing the cuboid in a separate, more distant, depth plane than the search array would effectively assign the former to the background, permitting contextual learning of only the item configuration in the foreground. Thus, on the foreground-learning account, contextual cueing was expected to be evident regardless of the change (removal) of the cuboid object in the test phase of Experiment 2. The block-retrieval account, by contrast, would predict diminished contextual cueing following the cuboid change (removal), as already seen in Experiment 1.

## Experiment 2

Experiment 2 was designed to examine whether a task-neutral context that is ‘segmented’ into the background by means of 3D depth cues would still be learned together with the item-based spatial context in the foreground during visual search. Previous work (Nakayama and Silvermann, 1986) has shown depth to be less costly than the other feature dimensions, such as color or motion, in search for a target defined by a cross-dimensional feature conjunction (e.g., searching for a white upward-moving target among black upward-moving and white downward-moving distractors). Targets ‘popped out’ of the display when they were defined by a conjunction of depth with color or depth with motion, but not when they were defined by motion and color. This suggests depth provides a stronger grouping or segregation cue than color or motion, efficiently guiding observers’ attention to the relevant sub-group (or depth plane) that contains target (while minimizing the interference from distractors in other depth planes). Thus, assuming that in Experiment 2, the task-neutral cuboid is effectively separated from the visual search plane, then, if contextual learning relies primarily on the foreground context, the cuboid should not be encoded into the learned, configural memory representation, and thus not interfere with contextual cueing when the cuboid is changed or removed. Otherwise, if foreground-background segmentation does not affect contextual learning, the findings should be similar to those of Experiment 1.

The method in Experiment 2 was essentially the same as in Experiment 1, except that the trial displays were now shown in 3D (using a 3D projector presentation system). The major difference relative to Experiment 1 was that the 12 search items were shown on the front and the cuboid on the back plane of a 3D (stereoscopic) search display (see **Figure 1B** for examples). Importantly, the display arrangements were the same as in Experiment 1 when viewed monocularly.

### Materials and Methods

#### Participants

As in Experiment 1, only participants who showed positive contextual cueing moved on to the test session. Sixteen participants took part in the initial training session (nine females, mean age: 25.13 ± 4.49) and 10 completed the subsequent test session (eight females, mean age 26.1 ± 5.36). They were paid by 8 € per hour for their participation.

#### Apparatus and Stimuli

The visual stimuli were presented via a 3D compatibility Optoma projector (HD131Xe) onto a white canvas at 120 Hz. Given that the 3D glasses (Optoma ZF2100) alternated the left and right shutters during the presentation, the frame rate for each eye was half of 120 Hz (i.e., 60 Hz). The experiment was conducted in a dimly lit cabin (0.12 cd/m^2^); the viewing distance was set at 77 cm, controlled by the use of a chin rest. The task-relevant (front) plane was defined by a transparent gray rectangle area (size 16° × 16°, luminance 56.78 cd/m^2^ RGB color = [128 128 128], see **Figure 1B**, right for examples), which enclosed the visual search items and the fixation cross. The back gray plane (RGB color = [128 128 128]) that contained the white cuboid was non-transparent. Hence, participants could view the back plane through the frontal (transparent) plane. Both depth planes were always available during a given trial (including the inter-trial interval) to enhance depth perception. The search displays comprised of one ‘T’ and eleven ‘L’s (size 0.8° × 0.8°, luminance 97.62 cd/m^2^) and were presented in the central area (size 13.2° × 13.2°) of the front plane. The cuboid (97.62 cd/m^2^) was presented approximately 6 cm behind the front plane when participants viewed the display with 3D ‘disparity’ glasses. Importantly, although the configural context and the cuboid were presented in different depth planes, the locations of the search items were aligned with the edges of the pseudo-cuboid when seen from participants’ viewpoint.

### Results

#### Search Task

Both the error rates and the proportion of outliers were low: mean error rates: 1.58% for all 16 participants, as well as for the selected ten positive cueing learners (The mean RT of the 10 positive cueing learners with epochs and contexts as factors are shown in **Figure 3B**); outliers: 2.98% for all 16 participants, 2.95% for the positive learners. A 2 (context: old, new) × 4 (epoch: 1–4) repeated-measures ANOVA on the error rates of the training session revealed no significant effects [context, *F*(1,15) = 0.01, *p* = 0.92, $\eta_{p}^{2}$ = 0.001; epoch, *F*(3,45) = 1.28, *p* = 0.29, $\eta_{p}^{2}$ = 0.08; interaction, *F*(3,45) = 0.33, *p* = 0.80, $\eta_{p}^{2}$ = 0.02], suggesting there was no improvement of performance accuracy over the course of training.

A repeated-measures ANOVA on the training-session RTs with context (old, new) and epoch (1–4) as factors revealed a significant main effect of epoch, though only a marginally significant effect of context: epoch, *F*(1.71, 25.63) = 18.74, *p* < 0.01, $\eta_{p}^{2}$ = 0.56 (RTs were 287 ms faster in epoch 4 than in epoch 1); context, *F*(1,15) = 4.22, *p* = 0.058, $\eta_{p}^{2}$ = 0.20 (RTs were 106 ms faster to old than to new displays); the context × epoch interaction was not significant, *F*(1.43, 21.48) = 0.78, *p* = 0.51, $\eta_{p}^{2}$ = 0.05. Further analyses suggested that contextual-cueing facilitation was relatively small in epoch 1 (59 ms) and larger from epoch 2 (>100 ms). When re-analyzing RTs from epochs 2 to 4 only (i.e., excluding epoch 1), the ANOVA revealed the main effect of context to be significant [*F*(1,9) = 18.51, *p* < 0.05, $\eta_{p}^{2}$ = 0.67]. Thus, taken together, contexts could be learned in a 3D scene, though the cueing effect was weaker compared to Experiment 1; this may suggest that the 3D display conditions made contextual learning somewhat more difficult. A breakdown of individual participants’ contextual gains is shown in **Figure 2B**. Six out of the sixteen participants (i.e., participant 12–16) failed to show positive contextual cueing in the whole training session, or in the last training epoch. This rate is in line with a previous study (Schlagbauer et al., 2012), in which some 30% of the participants (37.5% in the current experiment) failed to display positive or robust contextual cueing during the training stage.

Similar to Experiment 1, the ten participants who exhibited positive and robust contextual cueing went on to complete the test session, permitting the transfer effects of learned context to be examined under the cuboid-changed and, respectively, cuboid-removed conditions. Paired sample *t*-tests revealed significant contextual cueing facilitation in both epoch 5 [test session I: *t*(9) = 2.76, *p* < 0.05, mean effect of 308 ms] and epoch 6 [test session II: *t*(9) = 2.73, *p* < 0.05, mean effect of 217 ms]. In order to compare the magnitude of contextual cueing between the training and test sessions, we calculated contextual facilitation in the last two blocks of the training session, which revealed the cueing gains to be 361 ms, on average. Further paired-sample *t*-tests failed to reveal any significant difference in the magnitude of cueing gains between the last two training blocks and the two test sessions, in which the cuboid was either changed [*t*(9) = 0.62, *p* = 0.55, JZS Bayers factor = 3.59] or entirely removed [*t*(9) = 1.77, *p* = 0.11, JZS Bayers factor = 1.2]. The main finding of Experiment 2 thus contrasts with that of Experiment 1, consistent with the idea that when the cuboid can be effectively segmented as background, it will not be integrated into the configural representation underling contextual memory.

A further 2 (context: old, new) × 3 (experimental sessions: last two blocks of training session, test session I, test session II) repeated-measures ANOVA of the RTs revealed significant main effects of context and session, but the interaction was non-significant: context, *F*(1,9) = 21.08, *p* < 0.01, $\eta_{p}^{2}$ = 0.70; session, *F*(2,18) = 4.36, *p* < 0.05, $\eta_{p}^{2}$ = 0.33; interaction, *F*(2,17) = 0.89, *p* = 0.43, $\eta_{p}^{2}$ = 0.09. This effect pattern confirms that changes/removal of the cuboid on the back plane did not significantly impact contextual cueing. The session effect was mainly caused by the significant RT reduction from the training session (upward-pointing cuboid) to test session II (cuboid removed) [new: *t*(9) = 2.60, *p* < 0.05, mean effect of 283 ms; old: *t*(9) = 1.78, *p* = 0.11, mean effect of 110 ms]. There was no significant RT difference between the training session (with upward-pointing cuboid) and test session I (with downward-pointing cuboid) [new: *t*(9) = 0.43, *p* = 0.68, JZS Bayers factor = 3.94; old: *t*(9) = 0.36, *p* = 0.73, JZS Bayers factor = 4.05]. To summarize, manipulation in the test sessions, of the cuboid on separate depth plane to the search array did not affect acquired contextual cueing, even when the cuboid was removed entirely.

#### Recognition Performance

All trials were finished within the 20 s interval allowed. For the 10 participants who completed both the test and recognition sessions, the mean hit rates were 57.50, 46.25, and 46.25%, and the mean false alarm rates 50.00, 43.75, and 52.50% in the three recognition blocks (with upward-pointing, downward-pointing, and no cuboid), respectively. Paired sample *t*-tests revealed no significant differences between the hit and false alarm rates: upward-pointing cuboid, *t*(9) = 0.71, *p* = 0.50, JZS Bayes Factor = 3.41; downward-pointing cuboid, *t*(9) = 0.13, *p* = 0.76, JZS Bayes Factor = 4.72; no cuboid, *t*(9) = -1.00, *p* = 0.34, JZS Bayes Factor = 2.74. There was also a null-result when the hit and false alarm rates were collapsed across the three recognition blocks: *t*(9) = 0.22, *p* = 0.83, JZS Bayes Factor = 4.21. Also, there was no correlation between the overall (collapsed) recognition performance (d’) and the magnitude of contextual cueing in the last two test blocks: *r* = 0.04, *p* = 0.88. Taken together, these results favor the view that contextual cueing is an implicit memory effect.

### Discussion

In Experiment 2, the configural context and the task-neutral cuboid were separated on different depth planes. Although the search array was still overlaid on the edges of the cuboid, binocular cues (e.g., Nakayama and Silvermann, 1986; Nakayama et al., 1989) could be easily used for foreground-background segregation, assigning the cuboid to the background and thus decoupling it from contextual learning. The contextual cues thus acquired (over the course of training) were robust against any changes of the cuboid (in the test sessions), indicating that the learned configural context representation (underlying contextual cueing) did not include the task-neutral cuboid. This rules out the blocked-retrieval alternative, that is, the contextual cueing effect in Experiment 1 was affected by the variation of the – as such irrelevant – cuboid impeding the retrieval of the search-guiding contextual memory representation. Instead, the results are more in line with the segmentation-learning account, which proposes that information segmented to the foreground, including task-neutral object, is integrated into the contextual memory representation.

## Experiment 3

Note that the foreground-background segregation can also take place for the 2D visual information. To further corroborate the findings, we conducted a third experiment using normal 2D display (i.e., one depth plane), but varying the coupling between the position of the search items and the cuboid.

### Materials and Methods

The method was essentially the same as in Experiment 1, that both the configural context and the pseudo cuboid were presented on the same depth plane; no depth information was provided in this experiment. In order to achieve the pseudo cuboid as background task-neutral information, the search items were not constrained to the edges of the ‘upward-pointing’ and ‘downward-pointing’ cuboid shape, rather spread ‘off’ the cuboid (see **Figure 1** the bottom left, the 44 possible item locations inside the whole 13.2° × 13.2° square area). With such arrangement, there was no apparent spatial association between configural context and the ‘upward-pointing’ cuboid; thereupon the pseudo cuboid was likely to be segregated from the visual search items, becoming background task-neutral information. Same as in Experiment 2, we recruited in total 16 participants (eight females, mean age of 29.13 ± 4.60) in Experiment 3 with 15 (seven females, mean age of 29.33 ± 4.69) of them showed positive contextual cueing effect during early training session.

### Results

#### Search Task

Both the error rates and the ratio of outliers were low for all the 16 participants in the current experiment (mean error rates: 2.38%; outliers: 4.10%) as well as of the 15 positive learners (mean error rates: 2.15%; outliers: 3.92%). The error rates decreased significantly with epoch as main factor [*F*(5,75) = 3.58, *p* < 0.05, $\eta_{p}^{2}$ = 0.20] but comparable of context [*F*(1,15) = 3.64, *p* = 0.08, $\eta_{p}^{2}$ = 0.20] and interaction [*F*(5,75) = 0.39, *p* = 0.86, $\eta_{p}^{2}$ = 0.03] suggesting participant’s general accuracy improved regardless of display type. The overall results of the positive cueing learners are depicted in **Figure 3C**.

During the training session, repeated-measures ANOVA with context (old vs. new) and epoch (1–4) as factors of all the participants revealed significant main effects and interaction: epoch, *F*(15,45) = 26.02, *p* < 0.001, $\eta_{p}^{2}$ = 0.63, with 366 ms faster in epoch 4 compared to epoch 1; context, *F*(1,15) = 12.18, *p* < 0.01, $\eta_{p}^{2}$ = 0.49, with 187 ms faster for the old display compared to the new display; interaction between displays and epoch, *F*(3,45) = 4.38, *p* < 0.01, $\eta_{p}^{2}$ = 0.23. Further *post hoc* analysis suggested the significant interaction effect reflect a significant increase from the non-significant contextual cueing effect in epoch 1 [*t*(15) = -1.03, *p* = 0.32], to significant cueing in the following epochs (all *p’s* < 0.05). These results suggest both contextual cueing and procedural learning effect was manipulated during the training session for all the 16 participants in Experiment 3.

Similar as previous experiments, the fifteen participants who exhibited positive and robust contextual cueing went on to complete the test session, allowing the transfer effects of learned context to be examined under the cuboid-changed and, cuboid-removed conditions. Paired sample *t*-tests revealed significant contextual cueing facilitation in both epoch 5 [test session I: *t*(14) = 2.30, *p* < 0.05, mean effect of 277 ms] and epoch 6 [test session II: *t*(14) = 3.45, *p* < 0.01, mean effect of 212 ms]. In order to compare the magnitude of contextual cueing between the training and test sessions, we calculated contextual facilitation in the last two blocks of the training session, which revealed the cueing gains to be 293 ms, on average. Further paired-sample *t*-tests failed to reveal any significant difference in the magnitude of cueing gains between the last two training blocks and the two test sessions, in which the cuboid was either changed [*t*(14) = 0.26, *p* = 0.80, JZS Bayers factor = 4.98] or entirely removed [*t*(14) = 0.91, *p* = 0.37, JZS Bayers factor = 3.50].

A further 2 (context: old, new) × 3 (experimental sessions: last two blocks of training session, test session I, test session II) repeated-measures ANOVA of the RTs revealed significant main effects of context, but neither of session nor interaction: context, *F*(1,14) = 10.81, *p* < 0.01, $\eta_{p}^{2}$ = 0.44; session, *F*(2,28) = 1.59, *p* = 0.22, $\eta_{p}^{2}$ = 0.10; interaction, *F*(2,28) = 0.51, *p* = 0.61, $\eta_{p}^{2}$ = 0.04. This effect pattern confirms that changes/removal of the background cuboid did not significantly impact contextual cueing. Taken together, the main findings of Experiment 3, consistent with Experiment 2, further confirm the idea that when the cuboid can be effectively segmented as background, it is not integrated into the configural representation during contextual learning.

#### Recognition Results

All trials were finished within the 20 s. For the 15 participants who completed both the test and recognition sessions, the mean hit rates were 57.50, 56.67, and 48.33%, and the mean false alarm rates 44.17, 46.67, and 50.83% in the three recognition blocks with upward-pointing, downward-pointing, and no cuboid, respectively. Paired sample *t*-tests revealed no significant differences between the hit and false alarm rates of all the conditions: upward-pointing cuboid, *t*(14) = 0.16, *p* = 0.87, JZS Bayes Factor = 5.08; downward-pointing cuboid, *t*(14) = 0.67, *p* = 0.51, JZS Bayes Factor = 4.16; no cuboid, *t*(14) = -0.59, *p* = 0.57, JZS Bayes Factor = 4.43. There was also a null-result when the hit and false alarm rates were collapsed across the three recognition blocks: *t*(15) = 0.99, *p* = 0.34, JZS Bayes Factor = 3.26. Taken together, these results favor the view that contextual cueing is an implicit memory effect.

### Discussion

In Experiment 3, we weakened the explicit spatial association between the search array and the task-neutral cuboid on the same depth plane to examine whether the task-neutral context could be still involved in contextual learning. The results revealed that the learned contextual cueing was maintained regardless of any changes of the task-neutral context, which suggests that the task-neutral context (here the cuboid) was likely treated as background information, and not encoded into spatial contextual memory. Thus, the findings in Experiment 3 further confirmed our hypothesis that foreground-background segmentation influences contextual learning and retrieval, providing that a foreground task-neutral context could be learned together with the configural context (Experiment 1), but a background context was ignored during contextual learning (Experiments 2 and 3).

## General Discussion

The present study investigated foreground-background segmentation processes in contextually guided visual search. In three experiments, participants searched for a target in a display consisting of both the visual search items and an additional, task-neutral cuboid object. The location of the cuboid relative to the search items was systematically varied from being part of the foreground in Experiment 1 to being segmented into the background in Experiments 2 and 3. In the training session of Experiment 1, the search items were positioned on the edges of the cuboid within the same plane, such that all items were spatially linked to the cuboid object, following the Gestalt principle of uniform connectedness (Han et al., 1999). Given that uniform connectedness provides an effective means of perceptual grouping (Palmer, 1992; Palmer and Rock, 1994; Han et al., 1999), the search items and the task-neutral shape were expected to be grouped/integrated together, thus forming one ‘cuboid-like’ object – which is perceptually foregrounded and, thus, prioritized for attention (Nakayama et al., 1989; Mazza et al., 2005) and contextual learning (Jiang and Leung, 2005; Pollmann and Manginelli, 2009b). In Experiment 2, by contrast, the search items and the cuboid were presented in different depth planes (though with overlaid locations), that is, they were perceptually segregated and thus processed differently. In the last experiment, although presented on the same depth plane, the visual search items were randomly spread over the whole display except the edges of the cuboid, thus lacking any explicit connectedness on the visual stimuli. In the subsequent test sessions of all experiments, the cuboid was either rotated by 90° or removed, without any changes to the configural context of the (old) search arrays. Contextual cueing diminished in the test session of Experiment 1, but remained intact in Experiments 2 and 3. One major difference in the setup among three experiments was the relation between the cuboid and search items in terms of foreground vs. background. When the cuboid and the search items were perceived to be on the same depth plane in Experiment 1, they were both integrated in the same, learned contextual representation; however, when 3D depth cues automatically segregated the cuboid from the search items in Experiment 2, or when the search items were randomly presented on the display but not on the edges of the cuboids in Experiment 3, there were no interactions between cuboid and search items in contextual learning. Taken together, the findings highlight two important conclusions: (1) a ‘simple’ change of display properties from the training to the test sessions does not provide an adequate account of the pattern of (missing) transfer effects in Experiment 1 (blocked-retrieval alternative). (2) Rather, processes of foreground-background segmentation determine what is learned in contextual cueing (foreground-learning hypothesis).

Our set of findings bears directly on recent investigations of the cueing effect (for a review see Goujon et al., 2015). The critical issue is what type of information is learned in contextual cueing. A number of studies found that contextual associations are acquired (mainly) in the vicinity of the target and come subsequently to guide visual search (Olson and Chun, 2002; Kunar et al., 2006, 2008; Brady and Chun, 2007; Goujon et al., 2015). Other studies, by contrast, reported compelling evidence that contextual cueing involves associations between the target location and the entire distractor configuration and/or discrete distractor elements (i.e., global contextual cueing; (Jiang and Wagner, 2004; Beesley et al., 2015; Goujon et al., 2015). Our study might bridge this gap by showing that an initial, bottom-up-driven, foreground-background segmentation process modulates contextual learning. In other words, contextual cueing is developed based on the context in the focus of attention (such as the search items together with the task-neutral cuboid in Experiment 1). In addition to the visual search task, the results of the recognition tests argue in favor of learning being implicit in both experiments. While they do not definitely rule out some explicit component, they at least suggest that contextual cueing and explicit memory are, at most, weakly related.

Moreover, prioritization of foreground information in contextual learning may provide an explanation for many of the heterogeneous findings in the literature, including the aforementioned work on perceptual grouping (Jiang and Leung, 2005; Conci and von Muhlenen, 2009; Geyer et al., 2010; Conci et al., 2013) and the roles of configurations versus scenes in contextual cueing (Brooks et al., 2010; Kunar et al., 2013; Rosenbaum and Jiang, 2013). Concerning perceptual grouping: based on effective stimulus attributes, such as provided by binocular disparity (Palmer and Rock, 1994) or color (Jiang and Chun, 2001; Jiang and Leung, 2005), items in the search array could easily be grouped and segregated as foreground and background, respectively. Feature-based attention ensured that one group (e.g., the white items when the target was known to be white, as in Jiang and Leung, 2005) was prioritized (or ‘foregrounded’) for search, permitting contextual associations to be readily acquired from the repeated target-distractor configurations in this group. This would explain why a similar amount of contextual facilitation was observed between ‘attended old’ displays (in which only distractors sharing, say, the target color, appeared in repeated spatial arrangements) and ‘both old’ displays (in which all distractors, whether or not they shared the target color, appeared in repeated arrangements) – the explanation being that in both conditions, contextual learning took place for the prioritized item group. Consequently, when grouping occurs based on a less effective feature, such as size in the study of Conci and von Mühlenen (2011), contextual facilitation is reduced. Note that the size of the target (small vs. large) varied randomly from trial to trial, which would increase the chance of wrong segmentations, that is, observers might wrongly select the group that contains no target as foreground context, thus limiting the overall magnitude of contextual cueing.

In addition to perceptual grouping, foreground-background segmentation might also play a critical role in determining the interplay between scene- and configuration-based contextual cueing. For example, Kunar et al. (2013) first trained participants with old displays consisting of ‘predictable’ contexts of both a global color background and a search array (‘T’ and ‘L’s) in the learning session (i.e., if the target appeared at location X, the background color would be always ‘red’ and the location of the ‘L’s would be maintained constant). In the following test session, the search display changed into ‘color constant + varied array’ (i.e., the background color but not the search array predicted the target location) or ‘varied color + constant array’ (i.e., the search array but not the background color predicted the target location) conditions. Arguably, under these conditions, although the unique color background was predictive of the target location, it would nevertheless be segregated as background, because in everyday life spatial regularities are likely to be more important than color regularities (as colors are subject to change as a result of many factors, including illumination etc.). Consistent with this, Kunar et al. (2013) found that contextual cueing was strong in the ‘varied color + constant array’ condition, but weak in the ‘constant color + varied array’ condition. Such a ‘configuration-dominant’ effect was also reported by Brooks et al. (2010), who presented the search items on a ‘green table’ that was positioned in the center of a realistic scene display. This permitted the search items to be grouped together with the ‘green table’ and foregrounded, from the larger scene context that was segregated into the background. Note that the target in this study never appeared in the peripheral scene context. In contrast to Brooks et al. (2010), Rosenbaum and Jiang (2013) found a ‘scene-dominant’ effect, that is, the contextual cues acquired from repeated exposure to displays that were predictive in terms of both the search item configuration and the scene layout could only be transferred to the scene-predictive (configuration-varied), but not to the configuration-predictive (scene-varied) conditions. Note that in this study, the visual search items were distributed across the whole display (an area of 20° × 20° visual degrees), including both central and peripheral areas (whereas in Brooks et al. (2010); in addition, the search items were relatively small (i.e., one ‘T’ and eleven ‘L’s sized 0.56° × 0.56° visual degrees) and the inter-item distances were larger (i.e., the item presentation was less crowded) compared to Brooks et al. (2010) study. In this way, the configural context was physically and perceptually less salient than the predictive-scene context. In addition, natural scenes contain many spatial, temporal, and semantic (i.e., already acquired) regularities, making them a strong candidate for foreground segmentation and the learning of target-scene associations. Thus, to conclude: the present proposal of contextual learning (and, as a result, cueing) being constrained by foreground-background segmentation processes promises to provide a coherent, unified explanation of (seemingly) contradictory findings from previous studies regarding the relationship between scene- and configuration-centered contextual cueing.

To summarize, the present study systematically investigated the role of foreground-background segmentation for contextual cueing of visual search. The results revealed that information segmented as foreground information, even if this is task-neutral (i.e., irrelevant for deciding on the required response), determines what is learned and integrated in the search-guiding spatial context representation, as evidenced by interference when the task-neutral information changes during testing. This novel finding sheds light on the intrinsic contextual cueing mechanism and provides a possible answer as to why previous studies produced seemingly heterogeneous findings with regard to the type of information that is learned in contextual cueing.

## Author Contributions

Conception: XZ, ZS, TG, and HM. Experimental design: XZ, TG, and ZS. Data collection: XZ and LA. Data analysis: XZ and LA. Results interpretation: XZ, TG,LA, HM, ZS. Drafting: XZ and LA. Revision: ZS, TG, HM. Final Approval: HM. All authors agree to be accountable for the content of the work.

## Conflict of Interest Statement

The authors declare that the research was conducted in the absence of any commercial or financial relationships that could be construed as a potential conflict of interest.

## References

[B1] Annac E. Manginelli A. A. Pollmann S. Shi Z. Müller H. J. Geyer T. (2013). Memory under pressure: secondary-task effects on contextual cueing of visual search. *J. Vis.* 13 1–15. 10.1167/13.13.6 24190911

[B2] Baylis G. C. Driver J. (1992). Visual parsing and response competition: the effect of grouping factors. *Percept. Psychophys.* 51 145–162. 10.3758/BF03212239 1549433

[B3] Baylis G. C. Driver J. S. (1993). Visual attention and objects: evidence for hierarchical coding of locations. *J. Exp. Psychol. Hum. Percept. Perform.* 19 451–470. 8331310 10.1037//0096-1523.19.3.451

[B4] Beesley T. Vadillo M. A. Pearson D. Shan D. R. (2015). Pre-exposure of repeated search configurations facilitates subsequent contextual cuing of visual search. *J. Exp. Psychol. Learn. Mem. Cogn.* 41 348–362. 10.1037/xlm0000033 24999706

[B5] Brady T. F. Chun M. M. (2007). Spatial constraints on learning in visual search: modeling contextual cuing. *J. Exp. Psychol. Hum. Percept. Perform.* 33 798–815. 10.1037/0096-1523.33.4.798 17683229

[B6] Brockmole J. R. Castelhano M. S. Henderson J. M. (2006). Contextual cueing in naturalistic scenes: global and local contexts. *J. Exp. Psychol. Learn. Mem. Cogn.* 32 699–706. 10.1037/0278-7393.32.4.699 16822141

[B7] Brockmole J. R. Henderson J. M. (2006). Using real-world scenes as contextual cues for search. *Vis. Cogn.* 13 99–108. 10.1080/13506280500165188

[B8] Brooks D. I. Rasmussen I. P. Hollingworth A. (2010). The nesting of search contexts within natural scenes: evidence from contextual cuing. *J. Exp. Psychol. Hum. Percept. Perform.* 36 1046–1048. 10.1037/a0019257 PMC3163502 20731525

[B9] Chun M. M. (2000). Contextual cueing of visual attention. *Trends Cogn. Sci.* 4 170–178. 10.1016/S1364-6613(00)01476-5 10782102

[B10] Chun M. M. Jiang Y. (1998). Contextual cueing: implicit learning and memory of visual context guides spatial attention. *Cogn. Psychol.* 36 28–71. 10.1006/cogp.1998.0681 9679076

[B11] Conci M. Müller H. J. (2012). Contextual learning of multiple target locations in visual search. *Vis. Cogn.* 20 746–770. 10.1080/13506285.2012.694376

[B12] Conci M. Müller H. J. von Mühlenen A. (2013). Object-based implicit learning in visual search: perceptual segmentation constrains contextual cueing. *J. Vis.* 13 1–17. 10.1167/13.3.15 23838562

[B13] Conci M. Sun L. Müller H. J. (2011). Contextual remapping in visual search after predictable target-location changes. *Psychol. Res.* 75 279–289. 10.1007/s00426-010-0306-3 20725739

[B14] Conci M. von Muhlenen A. (2009). Region segmentation and contextual cuing in visual search. *Atten. Percept. Psychophys.* 71 1514–1524. 10.3758/APP.71.7.1514 19801612

[B15] Conci M. von Mühlenen A. (2011). Limitations of perceptual segmentation on contextual cueing in visual search. *Vis. Cogn.* 19 203–233. 10.1080/13506285.2010.518574

[B16] Driver J. Davis G. Russell C. Turatto M. Freeman E. (2001). Segmentation, attention and phenomenal visual objects. *Cognition* 80 61–95. 10.1016/S0010-0277(00)00151-7 11245840

[B17] Geringswald F. Baumgartner F. Pollmann S. (2012). Simulated loss of foveal vision eliminates visual search advantage in repeated displays. *Front. Hum. Neurosci.* 6:134 10.3389/fnhum.2012.00134 PMC3350129 22593741

[B18] Geyer T. Müller H. J. Assumpção L. Gais S. (2013). Sleep-Effects on implicit and explicit memory in repeated visual search. *PLoS ONE* 8:e69953 10.1371/journal.pone.0069953 PMC3732254 23936363

[B19] Geyer T. Shi Z. Müller H. J. (2010). Contextual cueing in multiconjunction visual search is dependent on color- and configuration-based intertrial contingencies. *J. Exp. Psychol. Hum. Percept. Perform.* 36 515–532. 10.1037/a0017448 20515186

[B20] Goujon A. Didierjean A. Thorpe S. (2015). Investigating implicit statistical learning mechanisms through contextual cueing. *Trends Cogn. Sci.* 19 524–533. 10.1016/j.tics.2015.07.009 26255970

[B21] Han S. Humphreys G. W. Chen L. (1999). Uniform connectedness and classical Gestalt principles of perceptual grouping. *Percept. Psychophys.* 61 661–674. 10.3758/BF03205537 10370335

[B22] Jeffries H. (1961). *Theory of Probability.* Oxford: Oxford University Press.

[B23] Jiang Y. Chun M. M. (2001). Selective attention modulates implicit learning. *Q. J. Exp. Psychol.* 54A, 1105–1124. 10.1080/713756001 11765735

[B24] Jiang Y. Leung A. W. (2005). Implicit learning of ignored visual context. *Psychon. Bull. Rev.* 12 100–106. 10.3758/BF03196353 15948286

[B25] Jiang Y. Wagner L. C. (2004). What is learned in spatial contextual cuing–configuration or individual locations? *Percept. Psychophys.* 66 454–463. 10.3758/BF03194893 15283070

[B26] Kunar M. A. Flusberg S. J. Wolfe J. M. (2006). Contextual cueing by global features. *Percept. Psychophys.* 68 1204–1216. 10.3758/BF03193721 17355043 PMC2678916

[B27] Kunar M. A. Flusberg S. J. Wolfe J. M. (2008). Time to guide: evidence for delayed attentional guidance in contextual cueing. *Vis. Cogn.* 16 804–825. 10.1080/13506280701751224 18846248 PMC2563807

[B28] Kunar M. A. John R. Sweetman H. (2013). A configural dominant account of contextual cueing: configural cues are stronger than colour cues. *Q. J. Exp. Psychol.* 67 1366–1382. 10.1080/17470218.2013.863373 24199842

[B29] Mazza V. Turatto M. Umilta C. (2005). Foreground-background segmentation and attention: a change blindness study. *Psychol. Res.* 69 201–210. 10.1007/s00426-004-0174-9 15597185

[B30] Nakayama K. Shimojo S. Silvermann G. H. (1989). Stereoscopic depth: its relation to image segmentation, grouping, and the recognition of occuluded objects. *Perception* 18 55–68. 10.1068/p180055 2771595

[B31] Nakayama K. Silvermann G. H. (1986). Serial and parallel processing of visual feature conjunctions. *Nature* 320 264–265. 10.1038/320264a0 3960106

[B32] Olson I. R. Chun M. M. (2002). Perceptual constraints on implicit learning of spatial context. *Vis. Cogn.* 9 273–302. 10.1080/13506280042000162

[B33] Palmer S. (1992). Common region: a new principle of perceptual grouping. *Cogn. Psychol.* 24 436–447. 10.1016/0010-0285(92)90014-S 1516361

[B34] Palmer S. Rock I. (1994). Rethinking perceptual organization: the role of uniform connectedness. *Psychon. Bull. Rev.* 1 29–55. 10.3758/BF03200760 24203413

[B35] Pollmann S. Manginelli A. A. (2009a). Anterior prefrontal involvement in implicit contextual change detection. *Front. Hum. Neurosci.* 3:28 10.3389/neuro.09.028.2009 PMC2764349 19844614

[B36] Pollmann S. Manginelli A. A. (2009b). Early implicit contextual change detection in anterior prefrontal cortex. [Research Support, Non-U.S. Gov’t]. *Brain Res.* 1263 87–92. 10.1016/j.brainres.2009.01.039 19368829

[B37] Rosenbaum G. M. Jiang Y. V. (2013). Interaction between scene-based and array-based contextual cueing. *Atten. Percept. Psychophys.* 75 888–899. 10.3758/s13414-013-0446-9 23572204

[B38] Rouder J. N. Speckman P. L. Sun D. Morey R. D. Iverson G. (2009). Bayesian t tests for accepting and rejecting the null hypothesis. *Psychon. Bull. Rev.* 16 225–237. 10.3758/PBR.16.2.225 19293088

[B39] Schlagbauer B. Müller H. J. Zehetleitner M. Geyer T. (2012). Awareness in contextual cueing of visual search as measured with concurrent access- and phenomenal-consciousness tasks. *J. Vis.* 12:25. 10.1167/12.11.25 23104818

[B40] Shi Z. Zang X. Jia L. Geyer T. Müller H. J. (2013). Transfer of contextual cueing in full-icon display remapping. *J. Vis.* 13 1–10. 10.1167/13.3.2 23444391

[B41] Treisman A. N. Gelade G. (1980). A feature-integration theory of attention. *Cogn. Psychol.* 12 97–136. 10.1016/0010-0285(80)90005-5 7351125

[B42] Vadillo M. A. Konstantinidis E. Shanks D. R. (2015). Underpowered samples, false negatives, and unconscious learning. *Psychon. Bull. Rev.* 23 87–102. 10.3758/s13423-015-0892-6 26122896 PMC4742512

[B43] Wolfe J. M. (2003). Moving towards solutions to some enduring controversies in visual search. *Trends Cogn. Sci.* 7:2 10.1016/S1364-6613(02)00024-4 12584025

[B44] Zang X. Jia L. Müller H. J. Shi Z. (2015). Invariant spatial context is learned but not retrieved in gaze-contingent limited-viewing search. *J. Exp. Psychol. Learn. Mem. Cogn.* 41 807–819. 25329087 10.1037/xlm0000060

[B45] Zellin M. Conci M. Von Mühlenen A. Müller H. J. (2013a). Here today, gone tomorrow - adaptation to change in memory-guided visual search. *PLoS ONE* 8:e59466 10.1371/journal.pone.0059466 PMC3598746 23555038

[B46] Zellin M. von Mühlenen A. Müller H. J. Conci M. (2013b). Statistical learning in the past modulates contextual cueing in the future. *J. Vis.* 13:19 10.1167/13.3.19 23881952

